# ESPB versus thoracic paravertebral block in pediatric nephrolithotomy: analytic population and inference after a negative superiority trial

**DOI:** 10.1016/j.bjane.2026.844849

**Published:** 2026-10-15

**Authors:** Swarupanjali Padhi, Prashant Ramdas Kokiwar, Janvi Patel, Ankita Kalra

**Affiliations:** a Noida Institute of Engineering & Technology (Pharmacy Institute), Department of Pharmaceutics, Greater Noida, India; b Malla Reddy Institute of Medical Sciences, Malla Reddy Vishwavidyapeeth, Department of Community Medicine, Telangana, India; c Dr. D. Y. Patil Vidyapeeth (Deemed-to-be-University), Hospital and Research Centre, Dr. D. Y. Patil Medical College, Maharashtra, India; d Graphic Era Hill University, Faculty of Pharmaceutical Sciences, Dehradun, India; e Graphic Era Deemed University, Centre for Promotion of Research, Dehradun, India

*Dear Editor*,

We read with interest the randomized, assessor-blind trial by Nabil et al. comparing erector spinae plane block (ESPB) with thoracic paravertebral block (PVB) in children undergoing percutaneous nephrolithotomy (PCNL). The study found no statistically significant between-group differences in time to first rescue nalbuphine or 24-hour rescue analgesic requirements, while ESPB took less time to perform.^1^ This is a clinically relevant question in pediatric urologic surgery.

The interpretation should remain aligned with the superiority design. Failure to demonstrate statistically significant superiority does not establish equivalence or non-inferiority, which require a prespecified clinical margin and an appropriate design and analysis. The confidence intervals reported for the primary outcome describe each group separately; reporting a between-group effect estimate with its confidence interval would better convey the precision of the comparison. Thus, the shorter performance time may inform procedural feasibility, but the absence of a significant analgesic difference should not be interpreted as proof of therapeutic comparability.

Clarification of the analytic population would further aid interpretation. One child allocated to ESPB was excluded after an intraoperative change in the surgical procedure, leaving 27 and 28 children in the respective analyses.^1^ If relevant outcome data are available, an intention-to-treat sensitivity analysis retaining the original allocation would help assess the robustness of the findings. If this is not feasible, the authors could explain what data were available and why the changed procedure precluded that analysis. Such clarification would allow readers to judge the implications of the exclusion without assuming that it materially altered the result.

The study defined block failure as requiring two doses of nalbuphine in the post-anesthesia care unit (PACU), and one child in each group received those doses.^1^ It would be helpful to state explicitly whether these children remained in the analyses, whether their first PACU rescue doses were counted as primary-outcome events, and whether both doses were included in the 24-hour rescue totals. Because block failure was defined using medication that also contributes to the efficacy endpoints, its handling can affect estimates of analgesic duration and rescue requirements. Reporting these details would distinguish outcomes following assignment to a block from outcomes restricted to successful blocks.

We also request clarification of the fentanyl dose cited as 1 mg.kg^-1^ in the version reviewed, which may represent a unit or typesetting discrepancy. Confirming the intended dose and ensuring consistent units across article formats would improve reproducibility.^1^

The wider evidence should also be interpreted according to its comparators and settings. A pediatric meta-analysis found no statistically significant differences between ESPB and caudal epidural block for selected analgesic outcomes, but this does not establish equivalence to thoracic PVB.^2^ A network meta-analysis in PCNL similarly found no significant differences among ESPB, PVB, and quadratus lumborum block for postoperative analgesic efficacy.^3^ Such findings cannot replace a pediatric equivalence or non-inferiority trial. In adults undergoing major oncological breast surgery, ESPB did not meet the non-inferiority criterion against PVB and provided less reliable dermatomal coverage.^4^ Although that result is not directly transferable to pediatric PCNL, it reinforces the need for procedure-specific inference. Transparent reporting of analysis populations, protocol deviations, and effect estimates would support interpretation, consistent with CONSORT 2025.^5^

We congratulate the investigators on addressing an important pediatric analgesia question. Clarifying the analysis population and rescue events, while interpreting the findings within the superiority design, would strengthen the clinical value of this trial without implying equivalence or non-inferiority.

## Data availability statement

No new data were generated or analyzed in this article. Data sharing is not applicable to this article.

## Funding

No specific funding was received for this work.

## Authors’ contributions

All authors contributed to the conception, drafting, critical revision, and final approval of this letter.

## Declaration of generative AI and AI-assisted technologies in the writing process

ChatGPT (OpenAI) was used to assist with language editing, manuscript polishing, and preparation of revisions responding to the editorial comments. The authors are responsible for the scientific interpretation, reference verification, and final approval of the manuscript.

## Conflicts of interest

The authors declare no conflicts of interest.
